# Role of integrins in the metastatic spread of high-grade serous ovarian cancer

**DOI:** 10.1007/s00404-021-06281-7

**Published:** 2021-10-24

**Authors:** Slavomir Krajnak, Jörg Jäkel, Katharina Anić, Roxana Schwab, Marcus Schmidt, Annette Hasenburg, Wilfried Roth, Walburgis Brenner, Marco Johannes Battista

**Affiliations:** 1 Department of Gynecology and Obstetrics, University Medical Center Mainz, Mainz, Germany; 2 Department of Pathology, University Medical Center Mainz, Mainz, Germany

**Keywords:** Integrin, High-grade serous ovarian cancer, Metastatic spread, Immunostaining, Prognosis

## Abstract

**Purpose:**

Integrins may be involved in the metastatic spread of high-grade serous ovarian cancer (HGSOC) which determines the therapeutical approach and prognosis. We investigated the integrin expression in primary tumor and metastases of advanced HGSOC.

**Methods:**

The expression of integrin α2, α4, α5, α6, and β1 was assessed by immunostaining in tumor samples of the ovary, omentum, and peritoneum of each patient. Differences in integrin expression among tumor localizations and their association with clinicopathological parameters were examined by Fisher’s exact test. The impact of integrin expression on progression-free survival (PFS) and overall survival (OS) was examined by Cox regression and Kaplan–Meier analyses.

**Results:**

Hundred and thirteen tumor samples of 40 HGSOC patients were examined. The expression of the integrins did not differ between the three tumor localizations (all *p* values > 0.05) with the exception of high expression of integrin α4 in primary tumor and omentum (52.5% versus 47.5%, *p* = 0.008) and primary tumor and peritoneum (52.5% versus 47.5%, *p* = 0.050). High expression of integrin α4 in peritoneum was associated with poorer PFS (HR 2.02 95% CI 1.01–4.05, *p* = 0.047), younger age (*p* = 0.047), and death (*p* = 0.046). Median PFS in patients with high expression of integrin α4 was 13.00 months, whereas median PFS in patients without high expression of integrin α4 was 21.00 months (*p* = 0.040). Expression of other integrins did not correlate with PFS or OS.

**Conclusion:**

Expression of integrin α4 may be altered during the metastatic spread of HGSOC and affect prognosis, whereas expression of integrin α2, α5, α6, and β1 did not reveal any prognostic value.

## Introduction

Ovarian cancer (OC) is one of the most commonly diagnosed cancers and the eighth leading causes of cancer-related death among women, accounting for about 295 thousand new cases and 185 thousand deaths in 2018, worldwide [1]. Despite the increasing survival rates for all cancers collectively, the mortality in OC has improved only slightly in recent decades with a 5-year survival rate well below 50% [2]. OC is a heterogeneous disease consisting of tumors differing in histopathology, immunochemistry, and molecular characteristics. High-grade serous ovarian cancer (HGSOC) is the most common histologic subtype, accounting for about 63% of epithelial OCs [3]. With the use of cytoreductive surgery and platinum-taxane-based chemotherapy, early stage disease is highly curable; however, the majority of patients presents with FIGO (Fédération Internationale de Gynécologie et d’Obstétrique) III/IV stage disease [2, 3]. Moreover, 70–80% of patients with advanced OC experience disease recurrence after initial therapy [2, 4]. Therefore, there is an urgent need to identify potential prognostic biomarkers and novel therapeutic options to improve the outcome of our patients with HGSOC.

Cells of ovarian surface epithelium or fallopian tube undergoing malignant transformation alter their adhesion properties during the process of epidermal–mesenchymal transformation (EMT), which in turn results in the shedding of tumor cells into the peritoneal cavity floating in the peritoneal fluids until they find a secondary attachment site for further growth [5, 6]. The surfaces of the peritoneal cavity, bowel, and omentum are the frequent sites for implantation of metastatic OC cells. The outer lining of these metastatic sites is comprised of a single layer of mesothelial cells, which express a variety of extracellular matrix (ECM) proteins, to which tumor cells can adhere before spreading [7, 8]. Integrins, binding ECM proteins, such as laminin, fibronectin, and collagen, are heterodimeric adhesion receptors expressed on the cell surface that consist of a α subunit and a β subunit. Thus far, 18 α subunits and 8 β subunits of integrins have been identified, forming 24 different integrin heterodimers with different specificities [9, 10]. Integrins generate an intracellular signal and, conversely, their functioning can be regulated by signals from inside the cell [9]. Integrin activation triggers a large variety of signal transduction events that modulate cell behaviors, such as adhesion, proliferation, survival or apoptosis, migration, and gene expression [11–14]. There are several studies, suggesting that integrins may play an important role in the metastatic spread of OC [11, 15, 16].

This study aimed to investigate the immunohistochemical expression profile of integrin α2, α4, α5, α6, and β1 at the primary tumor and the metastases of omentum and peritoneum in HGSOC patients and its prognostic value in the context of clinicopathological parameters, progression-free survival (PFS), and overall survival (OS).

## Materials and methods

### Patients and tissue samples

Patients with advanced HGSOC (FIGO IIIb-IV), who underwent primary surgery at our institution between 2004 and 2011, were included in the study, if paraffin-embedded tissue was available from the ovary and omentum or peritoneum. Clinicopathological and follow-up data until January 2019 were collected as previously reported by our group [17]. Tissue samples were provided by the tissue bank of the University Medical Center Mainz in accordance with the regulations of the tissue biobank and the approval of the Research Ethics Committee of the University Medical Center Mainz, Germany. For the analysis, all tissue samples were reassessed regarding histologic grade, histologic subtype, estrogen receptor (ER), progesterone receptor (PR), Ki-67, and p53 expression. Informed consent was obtained from all patients, and all clinical investigations were conducted according to the ethical and legal standards.

### Immunostaining

**Fig. 1:**
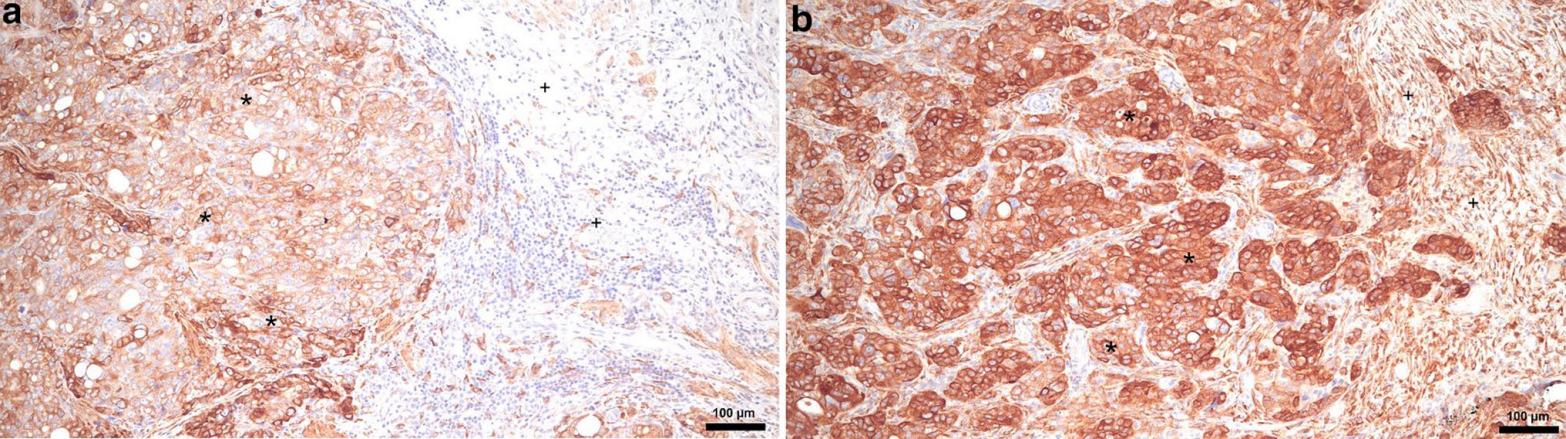
Strong immunostaining of integrin α4 in the primary tumor (**a**) and peritoneal metastasis of serous cancer (**b**). (*) Serous carcinoma cancer cells, (+) mesenchymal stromal cells

For immunostaining, 4-μm-thick formalin-fixed and paraffin-embedded tumor sections were stained with following primary integrin antibodies: α2, clone C-9, sc-74466; α4, clone A-7, sc-365209 (both Santa Cruz Biotechnology Inc., Heidelberg, Germany); α5, NBP1-84576 (Novus Biologicals Bio-Techne Ltd, Wiesbaden, Germany); α6, HPA012696 (Sigma-Aldrich Inc., Darmstadt, Germany); and β1, NBP2-16974 (Novus Biologicals Bio-Techne Ltd, Wiesbaden, Germany) according to the standard procedures. All the slides were analyzed using a Leica light microscope (Leica Microsystems Vertrieb Company, Wetzlar, Germany) by two of the authors (K.S. and J.J.). For the immunostaining analysis, we used a semiquantitative scoring method according to immunoreactive score (IRS) [18]. Each tissue sample was assessed by the intensity of immunostaining (0, negative; 1, weak; 2, moderate; 3, strong) (Fig. 1) and the area of positive cancer cells (0, negative; 1, 1–10%; 2, 11–50%; 3, 51–80%; 4, 80–100%). The final score was calculated by multiplying the scores for staining intensity and area of positive cells. Cases with score 0 were considered as negative, whereas cases with score ≥ 1 were considered as positive. In addition, scores from 0 to 4 were determined as low expression and scores from 5 to 12 as high expression.

### Statistical analyses

Statistical analyses were performed using the SPSS statistical software program, version 26.0 (SPSS Inc., Chicago, IL, USA). Patient characteristics were analyzed descriptively. Differences in integrin expression among tumor localizations and their association with clinicopathological parameters were determined by Fisher’s exact two-tailed test. The impact of integrin expression on PFS and OS was examined using Cox regression analyses and Kaplan–Meier estimator. In the Cox regression model, hazard ratio (HR) and 95% confidence interval (CI) was used. All tests were two-sided and a *p* value of < 0.05 was considered as statistically significant. As all analyses are explorative and not adjusted for multiple testing, the *p* values should be interpreted with caution and in connection with the effect estimates.

## Results

### Patient characteristics

**Fig. 2:**
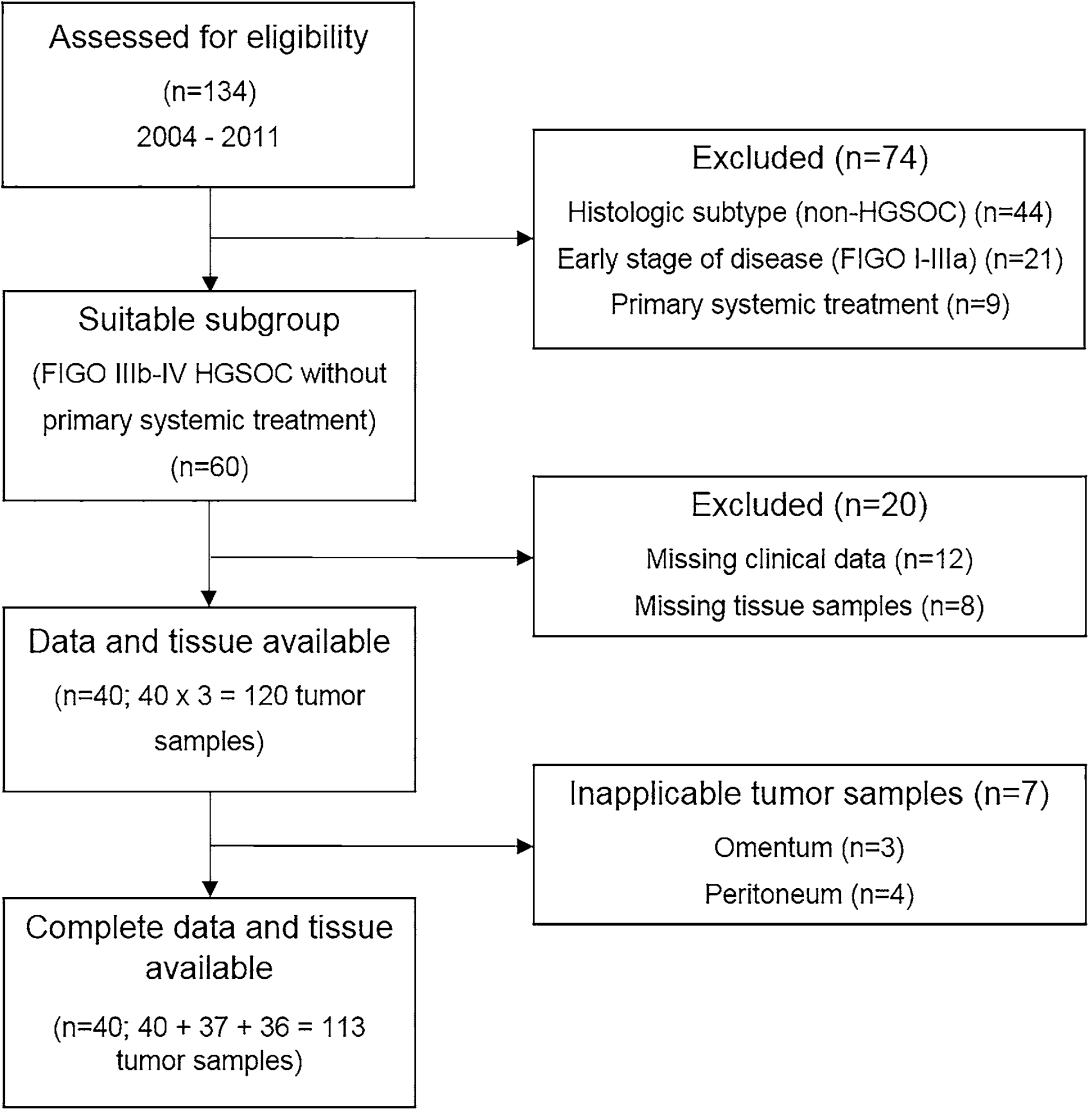
Patient enrollment. *FIGO* Fédération Internationale de Gynécologie et d'Obstétrique, *HGSOC* high-grade serous ovarian cancer

Between 2004 and 2011, a total of 134 patients with OC were screened in the Department of Gynecology and Obstetrics, University Medical Center Mainz, Germany (Fig. 2). 74 patients were excluded due to inappropriate histologic subtype, FIGO stage, or primary systemic treatment. 20 patients were excluded due to missing tissue samples or inappropriate follow-up information. Thereby, 40 advanced HGSOC patients with 113 tumor samples from the ovary, omentum, and peritoneum were analyzed. Patient characteristics are listed in Table 1. The median age was 63.9 years (range 31.7–78.3). At the time of first diagnosis, 31 (77.5%) patients presented FIGO III and 9 (22.5%) FIGO IV disease with a median tumor size of 5.0 cm (range 1.5–14.0). 10 (25.0%) patients were node-negative, 13 (32.5%) node-positive, and in 17 (42.5%) patients, lymph-node extirpation was not performed due to distant metastases (FIGO IV) or postoperative residual disease. Median Ki-67 expression was 50.0% (range 2.0–80.0). Complete surgical resection without macroscopic residual tumor was achieved in 15 (37.5%) patients. 27 (67.5%) patients completed adjuvant platinum-based chemotherapy; 7 (17.5%) patients terminated the therapy early due to occurrence of unacceptable toxicity or poor general condition. At the time of analysis, 4 patients were alive and 3 patients without recurrence. The median PFS was 15.00 months (95% CI 11.28–18.72); the median OS was 30.00 months (95% CI 25.35–34.65).

**Table 1:** Patient characteristics

Characteristics	Patients (*n* = 40) *n* (%)
Age (years)	
Median	63.9
Range	31.7–78.3
Primary tumor size (cm)	
Median	5.0
Range	1.5–14.0
Tumor stage (FIGO)	
I	0 (0.0)
II	0 (0.0)
III	31 (77.5)
IIIa	0 (0.0)
IIIb	4 (10.0)
IIIc	27 (67.5)
IV	9 (22.5)
Nodal status	
pN0	10 (25.0)
pN1	13 (32.5)
LNE not performed	17 (42.5)
Hormone receptor	
ER	
Positive	36 (90.0)
Negative	4 (10.0)
PR	
Positive	17 (42.5)
Negative	23 (57.5)
p53	
Positive	36 (90.0)
Negative	3 (7.5)
Missing	1 (2.5)
Ki-67 (%)	
Median	50.0
Range	2.0–80.0
Postoperative residual tumor burden	
Yes	25 (62.5)
No	15 (37.5)
Adjuvant chemotherapy	
Complete	27 (67.5)
Incomplete	7 (17.5)
Missing	6 (15.0)
Recurrence status	
Recurrence	37 (92.5)
Without recurrence	3 (7.5)
Living status	
Living	4 (10.0)
Dead	36 (90.0)

*ER* estrogen receptor, *FIGO* Fédération Internationale de Gynécologie et d’Obstétrique, *LNE* lymph-node extirpation, *PR* progesterone receptor

### Expression frequency of integrins

The expression rate of integrins ranged from 57.5% (integrin β1 in the omentum) to 95.0% (integrin α4 in the primary tumor), and did not differ between the three tumor localizations in most cases (*p* values > 0.05) (Table 2). A significant difference in the expression of integrin β1 was detected between primary tumor and omentum (77.5% versus 57.5%, *p* = 0.014). In addition, high expression of integrin α4 was observed less frequently in the omentum and peritoneum than in the primary tumor (both 47.5% versus 52.5%, *p* = 0.008; *p* = 0.050). High expression of integrin α2 was observed in 9 (22.5%), 4 (10.0%), and 4 (10.0%) cases in the primary tumor, omentum, and peritoneum, respectively (*p* = 0.244; *p* = 0.163). Integrin α5, α6, β1 were highly expressed in a maximum of 4 (10%) cases (integrin β1 in the peritoneum).

**Table 2:** Expression of integrin subunits regarding tumor localization

Localization	Primary tumor	Omentum	Peritoneum	*p* value Primary tumor/Omentum//Primary tumor/Peritoneum	
Integrin expression *n* (%)	Positivity//High expression	Positivity//High expression	Positivity/High expression	Positivity	High expression
α2	34 (85.0%)//9 (22.5%)	31 (77.5%)//4 (10.0%)	28 (70.0%)//4 (10.0%)	0.245//0.596	0.244//0.163
α4	38 (95.0%)//21 (52.5%)	35 (87.5%)//19 (47.5%)	34 (85.0%)//19 (47.5%)	0.107//1.000	0.008//0.050
α5	32 (80.0%)//1 (2.5%)	26 (65.0%)//1 (2.5%)	27 (67.5%)//2 (5.0%)	0.352//0.162	1.000//1.000
α6	31 (77.5%)//1 (2.5%)	33 (82.5%)//1 (2.5%)	35 (87.5%)//2 (5.0%)	0.198//1.000	1.000//1.000
β1	31 (77.5%)//0 (0.0%)	23 (57.5%)//1 (2.5%)	28 (70.0%)//4 (10.0%)	0.014//0.384	N/A//N/A

Regarding age, high expression of integrin α4 occurred more frequently in younger patients (omentum, *p* = 0.007; peritoneum, 0.047) (Table 3). Moreover, high expression of integrin α4 in peritoneum was observed more frequently in samples of deceased patients, whereas living patients did not show high expression of integrin α4 (*p* = 0.046). However, the associations of integrin α4 expression with recurrence status, residual tumor burden, tumor size, tumor stage, nodal status, hormone receptor status, Ki-67, and p53 expression were not statistically significant (Table 3). In integrin α2, α5, α6, and β1, Fisher’s exact two-tailed test revealed no significant associations between integrin expression and clinicopathological parameters (data not shown).

**Table 3:** Association between high expression of integrin α4 and the clinicopathological parameters

Localization		Primary tumor			Omentum			Peritoneum		
High expression		Yes	No	*p* value	Yes	No	*p* value	Yes	No	*p *value
Age (median; range)		60.1 (31.7–76.5)	65.5 (45.4–78.3)	0.180	59.1 (31.7–76.5)	69.8 (53.1–78.3)	0.007	61.5 (31.7–76.5)	67.5 (48.6–78.3)	0.047
Tumor size (median; range)		5.0 (1.5–14.0)	5.5 (2.0–12.0)	0.950	4.5 (1.5–14.0)	5.5 (2.0–12.0)	0.778	5.0 (1.5–11.0)	5.0 (2.0–14.0)	0.286
FIGO	III	14 (45.2%)	17 (54.8%)	0.133	13 (46.4%)	15 (53.6%)	0.252	13 (44.8%)	16 (55.2%)	0.232
	IV	7 (77.8%)	2 (22.2%)		6 (66.7%)	3 (33.3%)		6 (75.0%)	2 (25.0%)	
Nodal status	N0	5 (50.0%)	5 (50.0%)	0.685	4 (50.0%)	4 (50.0%)	0.608	3 (33.3%)	6 (66.7%)	0.387
	N1	8 (61.5%)	5 (38.5%)		6 (46.2%)	7 (53.8%)		7 (58.3%)	5 (41.7%)	
ER	Positive	19 (52.8%)	17 (47.2%)	1.000	17 (51.5%)	16 (48.5%)	0.677	18 (54.5%)	15 (45.5%)	0.340
	Negative	2 (50.0%)	2 (50.0%)		2 (50.0%)	2 (50.0%)		1 (25.0%)	3 (75.0%)	
PR	Positive	6 (35.3%)	11 (64.7%)	0.109	6 (37.5%)	10 (62.5%)	0.127	10 (62.5%)	6 (37.5%)	0.325
	Negative	15 (65.2%)	8 (34.8%)		13 (61.9%)	8 (38.1%)		9 (42.9%)	12 (57.1%)	
Ki-67 (median; range)		50.0 (2.0–80.0)	50.0 (15.0–80.0)	0.521	40.0 (5.0–80.0)	50.0 (2.0–80.0)	0.689	50.0 (2.0–80.0)	50.0 (5.0–80.0)	0.797
p53	Positive	18 (50.0%)	18 (50.0%)	1.000	16 (48.5%)	17 (51.5%)	0.500	18 (52.9%)	16 (47.1%)	1.000
	Negative	2 (66.7%)	1 (33.3%)		2 (66.7%)	1 (33.3%)		1 (50.0%)	1 (50.0%)	
Residual tumor	Yes	14 (56.0%)	11 (44.0%)	0.745	13 (56.5%)	10 (43.5%)	0.320	14 (60.9%)	9 (39.1%)	0.184
	No	7 (46.7%)	8 (53.3%)		6 (42.9%)	8 (57.1%)		5 (35.7%)	9 (64.3%)	
Recurrence status	Recurrence	20 (54.1%)	17 (45.9%)	0.596	18 (52.9%)	16 (47.1%)	0.479	19 (55.9%)	15 (44.1%)	0.105
	Without recurrence	1 (33.3%)	2 (66.7%)		1 (33.3%)	2 (66.7%)		0 (0.0%)	3 (100.0%)	
Living status	Living	2 (50.0%)	2 (50.0%)	1.000	1 (25.0%)	3 (75.0%)	0.281	0 (0.0%)	4 (100.0%)	0.046
	Dead	19 (52.8%)	17 (47.2%)		18 (54.5%)	15 (45.5%)		19 (57.6%)	14 (42.4%)	

*ER* estrogen receptor, *FIGO* Fédération Internationale de Gynécologie et d’Obstétrique, *PR* progesterone receptor

### Influence of integrins on survival

Cox regression analyses showed that integrin α4 expression in peritoneum significantly correlated with PFS. High expression of integrin α4 in peritoneum was associated with poorer PFS (HR 2.02 95% CI 1.01–4.05, *p* = 0.047). HR for OS was 1.93 (95% CI 0.96–3.89, *p* = 0.065). No correlations between high expression of integrin α4 and PFS or OS were observed in the primary tumor (PFS, HR 1.41 95% CI 0.74–2.70, *p* = 0.297; OS, HR 1.00 95% CI 0.52–1.95, *p* = 0.992) and omentum (PFS, HR 1.18 95% CI 0.60–2.33, *p* = 0.629; OS, HR 1.14 95% CI 0.57–2.27, *p* 0.712). Expression of integrin α2, α5, α6, and β1 did not correlate with PFS or OS (data not shown).

**Fig. 3:**
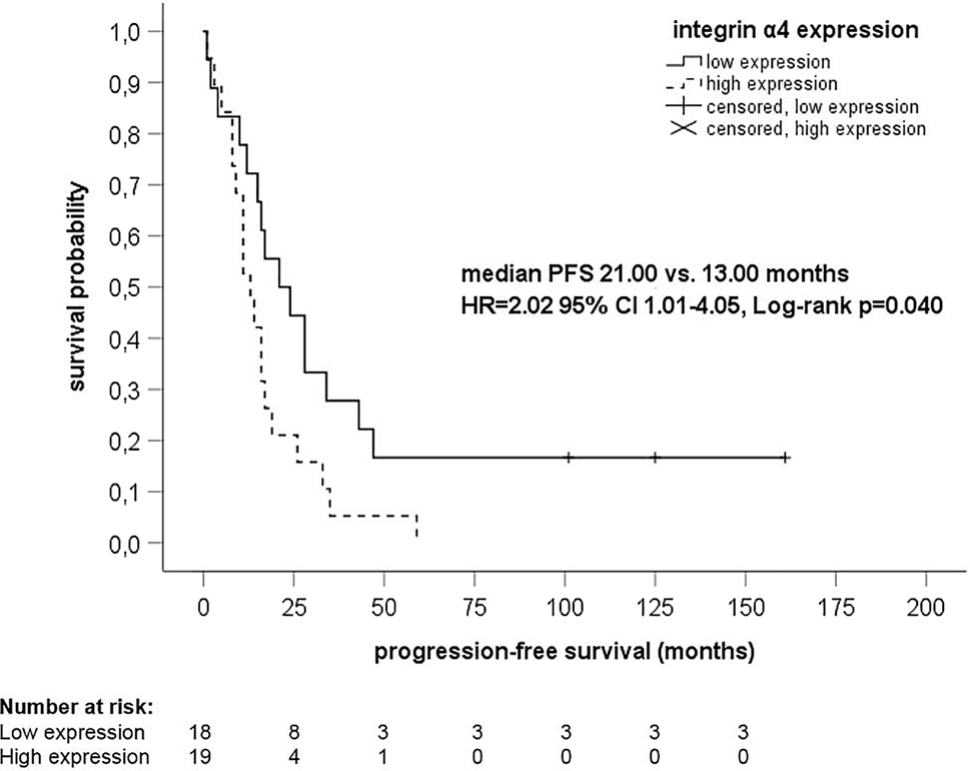
Kaplan–Meier analysis of progression-free survival regarding expression of integrin α4 in peritoneum. *CI* confidence interval, *HR* hazard ratio, *PFS* progression-free survival

**Fig. 4:**
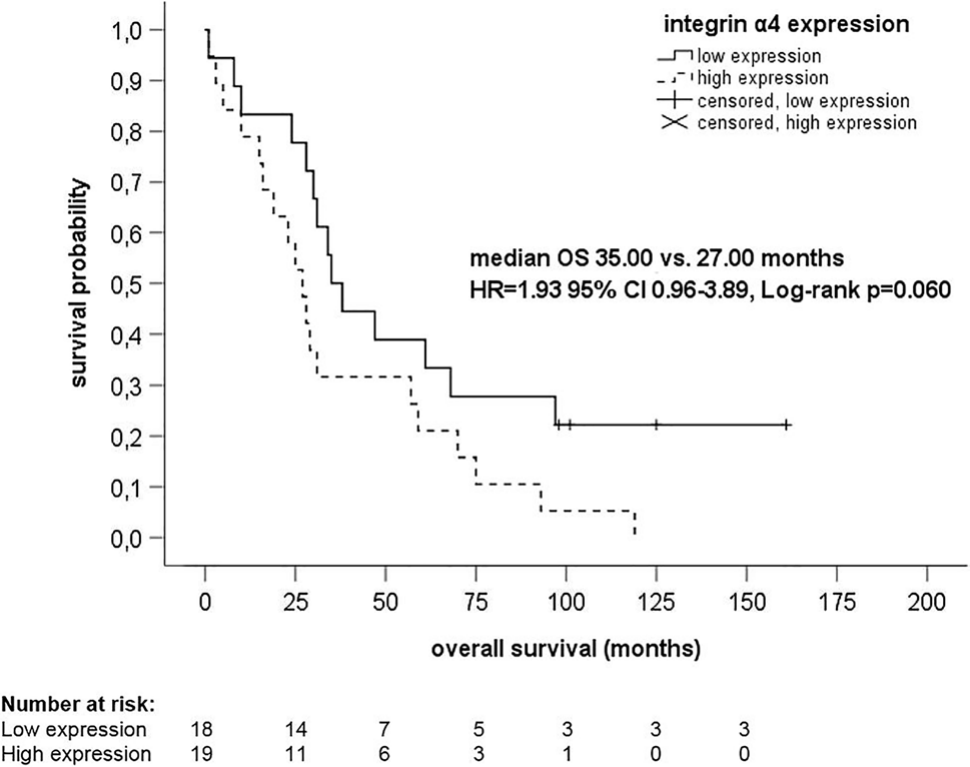
Kaplan–Meier analysis of overall survival regarding expression of integrin α4 in peritoneum. *CI* confidence interval, *HR* hazard ratio, *OS* overall survival

Kaplan–Meier analyses demonstrated significant associations between integrin α4 expression in peritoneum and PFS (*p* = 0.040) (Fig. 3), but not OS (*p* = 0.060) (Fig. 4). Median PFS in patients with high expression of integrin α4 in peritoneum was 13.00 months (95% CI 8.73–17.27); median PFS in patients without high expression of integrin α4 in peritoneum was 21.00 months (95% CI 6.45–35.55). Median OS in patients with high expression of integrin α4 in peritoneum was 27.00 months (95% CI 19.89–34.11); median OS in patients without high integrin α4 expression in peritoneum was 35.00 months (95% CI 26.68–43.32). However, the associations of high expression of integrin α4 with PFS and OS were not statistically significant in primary tumor and omentum.

## Discussion

In this explorative cohort study, we aimed to explore the role of various integrins in the metastatic spread in advanced HGSOC tumor samples. In most cases, the expression rate of integrins did not differ between the three tumor localizations and did not affect prognosis. However, integrin α4 appeared to be more frequently overexpressed in primary ovarian tumor samples than in omental and peritoneal metastases. Interestingly, the expression of integrin α4 may affect the prognosis, as shown in the Kaplan–Meier estimator and Cox regression analyses.

There are a plethora of studies investigating different integrins in OC. Contrary to our findings, Dötzer et al. [19] observed a high expression of integrin α2β1 in OC patients, which was identified as a marker for a poor prognosis with similar strength compared to FIGO stage and macroscopic residual tumor after surgery. High α2β1 expression in primary tumor was associated with a significant shorter PFS (*p* = 0.035) and platinum-free interval (*p* = 0.034). Remarkably, in this study, a α2β1 expression ≥ 20% was determined as high, whereas our scoring used IRS leading to a different classification. Shield et al. [8] showed that enhanced expression of α2β1 integrin in OC cell lines (HEY [HGSOC] and OVHS-1 [ovarian clear cell adenocarcinoma]) grown as spheroids may influence spheroid disaggregation and proteolysis responsible for the peritoneal dissemination of OC. In addition, α2β1 integrin was shown to promote OC cell invasion by increasing matrix metalloproteinase (MMP)-2/MMP-9 activation, thereby disaggregating tumor cell spheroids and enhancing cell proliferation [20]. Integrin α2β1 was also shown to be involved in induction of chemoresistance in OC via phosphatidylinositol 3-kinase (PI3K)/AKT signaling pathway [21]. Su et al. [22] isolated endothelial progenitor cells (EPCs) from OC patients, and demonstrated an increased integrin α4 expression, baseline migration, and adhesion mediated by the PI3K/AKT signaling pathway compared to those obtained from healthy subjects. Here, we reported for the first time that high expression of integrin α4 was significantly less frequent in the metastases of omentum and peritoneum than in the primary tumor. This may indicate that integrin α4 is involved in the metastatic spread of HGSOC. In addition, expression of integrin α4 showed associations with PFS, OS, and age at diagnosis. In particular, high expression of integrin α4 in peritoneum was significantly associated with poorer PFS and younger age. In a study with platinum-resistant mouse models, function-blocking antibodies directed against α4β1 sensitized advanced peritoneal disease to carboplatin and combination of integrin α4β1 blocking and carboplatin directly increased OC cell death [23]. Sawada et al. [24] evaluated the integrin α5 expression in 107 patients with FIGO II–IV advanced ovarian or peritoneal cancer. Each sample was scored based on the percentage of positive cells (0, ≤ 10%; 1, 10–25%; 2, 25–50%; 3, ≥ 50%) and the intensity of the staining (0, none; 1, weak; 2, strong). Only the samples which had strong staining of integrin α5 in ≥ 50% of tumor cells were considered as tumors overexpressing α5 integrin. In 9% of patients (7% of HGSOC patients), overexpression of integrin α5 was detected with a median OS of 26 months versus 35 months in patients with low or negative integrin expression (*p* = 0.03). However, in our study, high expression of integrin α5 was detected only in one sample (2.5%) of the primary tumor and omentum, respectively, and two samples (5.0%) of peritoneum, and did not correlate with PFS or OS. In a study by Wei et al. [25], expression of integrin α6 was in tissues of chemoresistant OC patients higher than in those of chemosensitive OC patients (60.0% versus 31.0%, p < 0.05). The intensity of immunostaining was graded as follows: 0, weak; 1 + , moderate; 2 + , strong; and 3 + , very strong. The area of positive cancer cells was categorized as follows: 1 + , 0–10%; 2 + , 11–50%; 3 + , 51–75%; and 4 + , 75–100%. The score for each section was calculated by multiplying the scores for both the staining intensity and the area of positive cells. Scores of 0–3 were designated as low expression; scores of 4–12 were designated as high expression. Kaplan–Meier analyses revealed a significantly poorer OS (*p* = 0.0008, HR 0.83 95% CI 0.73–0.95), but not PFS (*p* = 0.30, HR 0.93 95% CI 0.82–1.06) associated with high integrin α6 expression in a cohort of 1,583 OC cases. In the present study, we used a similar scoring system and observed the expression of integrin α6 between 77.5 and 87.5%. Despite this fact, we could not detect any correlations between integrin α6 expression and PFS or OS.

Taken together, several studies demonstrated that integrins may play an important role in the metastatic spread of OC. However, there is no convincing evidence of associations between integrins and prognosis in OC. Comparison of immunohistochemical studies is difficult, because different scoring systems and thresholds of high expression are used. Moreover, larger metastases may have already overgrown the primary site of cancer and peritoneal cell contact, limiting the assessment of the role of integrins at certain points in time.

A weakness of our study is the retrospective design; thus, the interpretation of the presented results is limited. However, a strength is that we assessed the expression of integrins within a very homogenous group of advanced HGSOC in three different tumor localizations, allowing us to obtain a comprehensive picture of the spatial role of integrins in HGSOC.

In conclusion, the present study showed that expression of integrin α4 may be altered during the metastatic spread of HGSOC and affect prognosis. Moreover, we demonstrated that high expression of integrin α4 in peritoneum may be prognostically relevant. Expression of integrin α2, α5, α6, and β1 did not reveal any prognostic value in HGSOC. These novel findings support further efforts to investigate α4 integrin in HGSOC.

## Data Availability

The datasets generated during the current study are available from the corresponding author on reasonable request.
